# Explainable AI for CHO cell culture media optimization and prediction of critical quality attribute

**DOI:** 10.1007/s00253-024-13147-w

**Published:** 2024-04-24

**Authors:** Neelesh Gangwar, Keerthiveena Balraj, Anurag S. Rathore

**Affiliations:** 1 School of Interdisciplinary Research, Indian Institute of Technology, Delhi, New Delhi, 110016 India; 2 Yardi School of Artificial Intelligence, Indian Institute of Technology, Delhi, New Delhi, 110016 India; 3 Department of Chemical Engineering, Indian Institute of Technology, Delhi, New Delhi, 110016 India

**Keywords:** Biosimilar, Charge variants, Media development, Feature ranking, Feature selection, Machine learning

## Abstract

**Abstract:**

Cell culture media play a critical role in cell growth and propagation by providing a substrate; media components can also modulate the critical quality attributes (CQAs). However, the inherent complexity of the cell culture media makes unraveling the impact of the various media components on cell growth and CQAs non-trivial. In this study, we demonstrate an end-to-end machine learning framework for media component selection and prediction of CQAs. The preliminary dataset for feature selection was generated by performing CHO-GS (-/-) cell culture in media formulations with varying metal ion concentrations. Acidic and basic charge variant composition of the innovator product (24.97 ± 0.54% acidic and 11.41 ± 1.44% basic) was chosen as the target variable to evaluate the media formulations. Pearson’s correlation coefficient and random forest-based techniques were used for feature ranking and feature selection for the prediction of acidic and basic charge variants. Furthermore, a global interpretation analysis using SHapley Additive exPlanations was utilized to select optimal features by evaluating the contributions of each feature in the extracted vectors. Finally, the medium combinations were predicted by employing fifteen different regression models and utilizing a grid search and random search cross-validation for hyperparameter optimization. Experimental results demonstrate that Fe and Zn significantly impact the charge variant profile. This study aims to offer insights that are pertinent to both innovators seeking to establish a complete pipeline for media development and optimization and biosimilar-based manufacturers who strive to demonstrate the analytical and functional biosimilarity of their products to the innovator.

**Key points:**

*• Developed a framework for optimizing media components and prediction of CQA.*

*• SHAP enhances global interpretability, aiding informed decision-making.*

*• Fifteen regression models were employed to predict medium combinations.*

**Supplementary Information:**

The online version contains supplementary material available at 10.1007/s00253-024-13147-w.

## Introduction

Cell culture media development and optimization is one of the critical and resource and time-intensive activities that are performed by all biopharmaceutical manufacturers. The primary challenge that is faced comes from the fact that both the media and the mammalian cells are highly complex systems. Cell culture media consist of hundreds of components, such as amino acids, fatty acids, vitamins, trace elements, and salts, and all of these could potentially impact cell growth, protein production, as well as the critical quality attributes (CQA) of the therapeutic product (Ritacco et al. 2018; Combe and Sokolenko 2021). These impacts, not surprisingly, have been a topic of investigation by numerous researchers (Zhou et al. 2010; Kaschak et al. 2011; Xu et al. 2018; Gangwar et al. 2021; Graham et al. 2021).

Various approaches have been used to optimize cell culture media components. These include model-based (Kotidis et al. 2019), feeding-based (Sun et al. 2013), and metabolic flux-based (Xing et al. 2011). Investigating media components via conventional one-factor-at-time (OFAT) (Hong et al. 2014) or two-factor (Sun et al. 2013; Radhakrishnan et al. 2018; Polanco et al. 2023) methods is time- and resource-consuming. Lately, statistical approaches such as the design of experiments (DOE) and multivariate data analysis (MVDA) (Salim et al. 2022) have gained popularity but do suffer from shortcomings such as the limitation on the maximum number of components that can be experimentally examined via a DOE and use of quadratic polynomial approximation, which may be too simple to represent the comprehensive interactions between the medium and the cell.

The last few years have witnessed an increasing application of machine learning (ML) approaches to deal with the amount and intrinsic complexity of biological data (Puranik et al. 2022; Yang et al. 2023; Rathore et al. 2023). The typical process involves handling input data, training the fundamental model, and making predictions. The cell culture medium serves as an excellent example of a well-ordered dataset, frequently including several components functioning as variable features. Feature selection, prediction, and optimization all play significant roles in medium development (Zhou et al. 2023). ML-based approaches have been demonstrated to have been successfully applied for medium development (Hashizume and Ying 2023) for T-cells (Grzesik and Warth 2021), cyanobacteria culture (Havel et al. 2006), as well as for HeLa-S3 cell lines (Hashizume et al. 2023).

In this study, we demonstrate how ML can be effectively used for feature selection, CQA prediction, and medium optimization. Metal ions were largely shown to modulate charge variants, particularly Fe (Chung et al. 2019), Cu (Kaschak et al. 2011), and Zn (Luo et al. 2012; Graham et al. 2020). Charge variants are believed to impact the efficacy of the biotherapeutic product and hence biosimilar manufacturers strive to match the charge variant composition of the innovator product (Khawli et al. 2010). ML was applied to the preliminary dataset to first identify the metal ions that exhibit significant impact by feature ranking method (Chicco and Rovelli 2019; Chicco and Jurman 2020). The primary emphasis of this study was on the utilization of transparent ML algorithms (white box), including linear regression, lasso regression, ridge regression, lasso least angle regression, Bayesian ridge, decision tree regressor, Huber regressor, and support vector machine. Additionally, some complex (black box) and less interpretable models, such as random forest regressor, CatBoost regressor, extreme gradient boosting, gradient boosting regressor, elastic net, extra tree regressor, and *K* neighbors regressor, were also examined. Both types have their advantages with respect to prediction and interpretability. Black box algorithms are good in prediction while others are good in interpretation.

## Material and methods

**Fig. 1:**
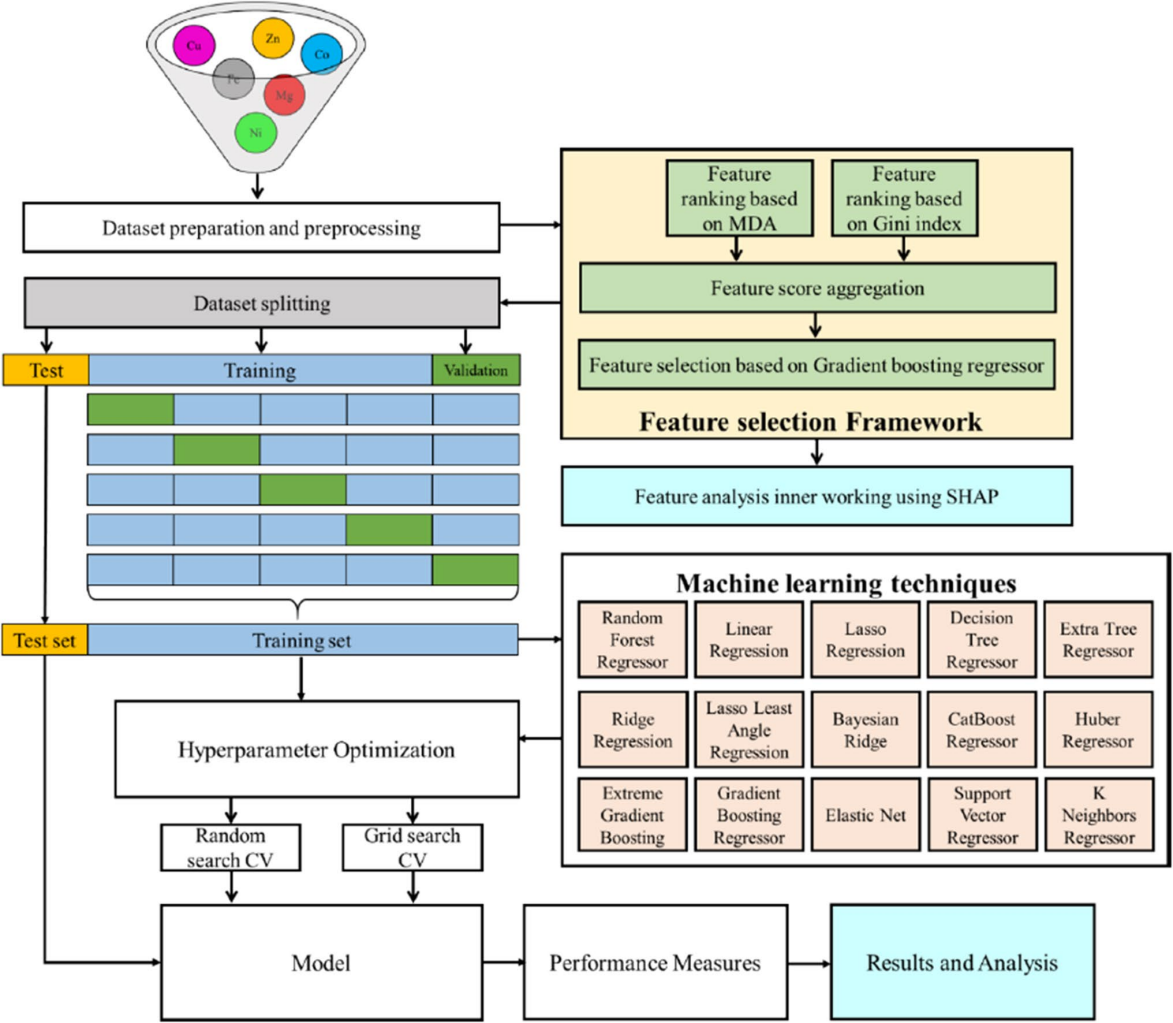
Proposed machine learning framework for prediction of critical quality attributes

In this study, a hybrid machine learning framework is proposed to optimize CHO cell culture media and predict the critical quality attribute. The model is composed of machine learning techniques including random forest regression, linear regression, lasso regression, decision tree regression, extra tree regression, ridge regression, lasso least regression, Bayesian ridge, catboost regression, Huber regression, extreme gradient boosting, gradient boosting regression, elastic net, support vector regression, and *k*-nearest regression. A graphical representation of the proposed framework is provided in Fig. 1. The pipeline consists of five distinct operational stages: (1) preparation and preprocessing, (2) feature selection and analysis, (3) optimization, (4) model development, and (5) model evaluation. Media formulations were prepared by supplementing the various combinations of metal salts such as copper (Cu), iron (Fe), zinc (Zn), manganese (Mn), magnesium (Mg), cobalt (Co), and nickel (Ni) into the basal medium. To enhance the accuracy of the proposed algorithm, it is necessary to perform preprocessing steps on the dataset, including data sampling, missing value imputation, and normalization. Following the preprocessing stage, two distinct approaches, namely, mean decrease accuracy (MDA) and Gini Index, were used, and SHapley Additive exPlanations were utilized to visualize the significance of the features. For training the model, the dataset is divided into *K* equal parts (*K* =  6), and the model that is trained is verified by utilizing the remaining dataset. Following the data pre-processing, various models for machine learning are established utilizing the hyperparameter optimization approach with cross-validation. Hyperparameter tuning methods for finding the optimal values for a model’s parameters include grid search and random search. Finally, a total of fifteen ML methods were used to screen metal ion concentrations, analyze their impact on the charge variant profile, and estimate the optimal concentrations.

### Cell line and reagents

IgG1 protein (Trastuzumab) producing CHO-GS(-/-) cell line was used and was provided by an industrial collaborator (Imgenex®, Bhubaneswar, India). The cell line is suitable for growth in glutamine-free media, once transfected with the vector containing glutamine synthetase gene (GS). A commercially available proprietary cell culture medium CD CHO® (Gibco™—12,490–001) was used as a basal medium, and Efficient Feed B medium (Gibco™—A1245605) as a medium feed supplement for fed-batch culture. Metal salts, copper (II) chloride dihydrate, magnesium sulfate heptahydrate, manganese chloride, iron sulfate heptahydrate, cobalt sulfate heptahydrate, zinc chloride, and nickel (II) chloride hexahydrate were purchased from Merck (Kenilworth, NJ, USA).

### Cell culture

Working cell bank (WCB) vial was revived from liquid nitrogen (− 196 °C) for 2 min at 37 °C in the water bath. As cells were thawed, immediately transferred into a freshly prepared CD CHO basal medium. Cells were passaged and maintained at least 2–3 times in basal media in a shaker flask (SF) after being well acclimated before the main experiment. Cell culture propagated in 125-mL shaker flasks (SF-125) having a working volume of 30 mL was incubated in a humidified incubator shaker (New Brunswick™ S41i—Eppendorf), at 5% CO_2_ concentration at 37 °C and 110 rpm agitation. Culture flasks were seeded with the initial cell density of 0.5 × 10^6^ cells/mL and were in both batch and fed-batch mode.

Batch mode was operated for 6 days, and these data were utilized for feature selection and regression model development while fed-batch data were used for validation of optimized media with respect to the control (basal medium). Fed-batch mode operated for 10 days with 10% of efficient feed B supplemented every alternate day starting from day 3rd to day 9th. Samples were collected every alternate day before the addition of feed and analyzed for various culture metabolites, cell count, and viability. Flasks were harvested on day 6 of culture for batch and day 10 for fed-batch, after centrifugation (Eppendorf – 5810R) at 5000 rpm and 25 °C. Harvest (spent medium) was further analyzed for titer, charge variants, and low and high molecular variants.

Trypan Blue exclusion method was used with a hemocytometer to determine cell viability and count. This device is based on the cell membrane’s structural integrity. In contrast to dead cells, which stain and appear dark, living cells have a well-integrated membrane that prevents dye from entering and prevents staining (Strober 2001).

### Medium formulations

Media formulations were prepared by supplementing the various combinations of metal salts into the basal medium. Previous studies have also demonstrated the significance of metal ions in determining the CQAs. Concentrations of the metal ions (Cu, Fe, Zn, Mn, Mg, Co, and Ni) were based on previous literature (Gangwar et al. 2021), shown in parts per million (ppm) and summarized in Table 1.

**Table 1:** Summary of the dataset used in this study

Features	Abbreviations	Variable type	Measurements	Range
Copper	Cu	Continuous [X1]	ppm	[0, 1, 2]
Iron	Fe	Continuous [X2]	ppm	[1, 13, 25]
Zinc	Zn	Continuous [X3]	ppm	[0.39, 5.2, 10.0]
Manganese	Mn	Continuous [X4]	ppm	[0.05, 0.78, 1.5]
Magnesium	Mg	Continuous [X5]	ppm	[19.5, 39.75, 60.00]
Cobalt	Co	Continuous [X6]	ppm	[0.06, 1.03, 2.00]
Nickel	Ni	Continuous [X7]	ppm	[0, 1, 2]
Acidic charge variant (target)	Acidic	Continuous [Y1]	Percentage (%)	[16.3–28.66]
Basic charge variants (target)	Basic	Continuous [Y2]	Percentage (%)	[10.06–14.5]

### Analytical methods

Culture harvest was purified by preparative Protein A chromatography using an Akta Avant (GE Healthcare, Sweden) system. Mobile phases consisted of equilibration buffer (50 mM phosphate and 150 mM NaCl, pH 7.5) and elution buffer (100 mM glycine, pH 3.0). The purification protocol was followed as per the established procedure (Rathore and Narnaware 2022).

Protein A eluted fractions were further analyzed for charge heterogeneity using cation-exchange high-performance liquid chromatography (CE‐HPLC). The analysis was performed on a Thermo Fisher RSLC system (Thermo Fisher Scientific, Waltham, 8 USA), having a DAD detector. The CEX method used to evaluate charge variants consisted of a linear gradient of mobile phase A (15 mM sodium phosphate, pH 6.2) and mobile phase B (150 mm sodium phosphate, pH 6.2) with 0.05% sodium azide. The separation was performed on a MAbPaC SCX‐10RS column (4.6 × 250 mm, Thermo Fisher Scientific, Waltham, USA) at a flow rate of 0.8 mL/min and 28 °C, and elution was monitored using UV absorbance at 280 nm. The reference CEX profile is provided in Fig. S1.

For estimating metal ions, inductively coupled plasma mass spectrometry (ICP-MS, Agilent Technologies, USA) was used. It combines a high-temperature ICP source with a mass spectrometer. The ICP ionizes the atoms of the elements in the sample. These ions are then separated and detected by the mass spectrometer.

### Dataset

The screening dataset was generated by culturing cells in batch mode using various media formulations and prepared as suggested in the previous section. Charge variants, i.e., acidic (*Y*_1_) and basic (*Y*_2_) variant amounts in purified mAb were taken as target variables. Media formulations having different concentrations of metal ions were used as predictor variables. A total of seven metal ions were taken as features or predictors, while a total of 34 formulations used in the experiment were used as observations. Finally, the features with the corresponding targets were fed as an input to the feature selection framework. The dataset (Table S1) and variables are explained in Table 1.

### Machine learning methods

Feature selection based on various biostatistics tools and ML approaches were discussed in order to identify the features that have significant effects on the target variables. Then, medium optimization was performed to get the optimum concentration of Fe and Zn to achieve the desired charge variant profile. Finally, different ML regressor models on featured variables were used to evaluate the performance of various models for the prediction of charge variants.

### Feature ranking

The possibility of a feature being connected to the target variable was effectively quantified by statistical methods, with the coefficients produced by the Pearson correlation coefficient (PCC). Using these scores, we were able to construct a ranking of the features, which is based on their degree of association with the target variable. Features with higher scores indicate a stronger relationship with the target, while those with lower scores exhibit a weaker connection (Sedgwick 2012; Obilor and Amadi 2018). This approach allows us to effectively identify and prioritize features based on their relevance to the target variable. Pearson correlation matrix was generated using the “seaborn (version: 0.12.2),” a Python library.

For ML feature ranking, we focused on embedded methods such as random forests and gradient-boosting regressors. The “RandomForestRegressor” and “GradientBoostingRegressor” functions from the “ensemble” module of the “scikit-learn (version:1.3.0)” library were used respectively for ML-based feature selection. Random forests provides two feature ranking techniques: permutation-based feature importance or mean decrease in accuracy (Altmann et al. 2010) (“permutation_importance” function in the “inspection” module of the “scikit-learn” library) and Gini importance or mean impurity reduction (Menze et al. 2009; Nembrini et al. 2018) (inbuilt attribute “feature_importances” in “RandomForestRegressor” function of “ensemble” module in “scikit-learn” library). SHAP (SHapley Additive exPlanations) is a game theoretic approach to explain the output of any machine learning model (Lundberg and Lee 2017). We have used SHAP to explain the contribution of features.

### Model development

Supervised learning models were developed for the prediction of charge variant composition of mAbs produced in various media formulations after the culturing of cells. Several ML techniques such as linear regression (LR), lasso regression, ridge regression, lasso least angle regression (LLA), Bayesian ridge (BR), decision tree regressor (DT), Huber regressor, support vector machine (SVR), random forest regressor (RF), CatBoost regressor, extreme gradient boosting (XGBoost), gradient boosting regressor (GBR), elastic net, extra tree regressor (ET), and *K* neighbors regressor (KNN) were employed.

### Medium optimization

One of the ML-based boosting techniques, the gradient-boosting regressor (GBR), was used to optimize media components. The “GradientBoostingRegressor” from the “ensemble” module of the “scikit-learn” library was used to construct the ML model, where the medium components and charge variants were employed as the explanator and the objective variables, respectively. Fivefold cross-validation was performed to search for hyperparameters using both grid and randomized search. “GridSearchCV” in the “model_selection” module of the “scikit-learn” library was used for grid search of hyperparameter tunning while “RandomizedSearchCV” of the same module and library was used for randomized search. The hyperparameters were searched for “learning_rate” from 0.001 to 0.5 in increments of 0.005, “max_depth” from 2 to 5 in increments of 1, and n_estimators at 300 and 400, respectively. The other hyperparameters were used by default.

### Performance measure

To evaluate prediction accuracy, various metrics were used, including mean absolute error (MAE), mean squared error (MSE), root mean square error (RMSE), coefficient of determination (*R*-sqr), and adjusted *R*-squared (Adj *R*-sqr). The “mean_absolute_error,” “mean_squared_error,” and “r2_score” functions from the “metrics” module within the “scikit-learn” library were employed to calculate mean absolute error (MAE), mean squared error (MSE), and coefficient of determination (*R*^2^), respectively. Root mean squared error (RMSE) was computed by calculating the square root of MSE using the “sqrt” function from the “numpy” library. Adjusted *R*^2^ (Adj_*R*^2^) was calculated by using *R*^2^, number of features (*k*), and number of observations (*n*) using the following formula:

$${{\text{Adj}}\_R}^{2}=1- \frac{(1-{r}^{2})(n-1)}{(n-k-1)}$$

A prediction accuracy assessment of the machine learning models was conducted through a sixfold cross-validation approach.

## Results

Datasets were analyzed, and models were developed using Python (version: 3.9.10). Excel (version 2309, Microsoft Office 365) and Origin® were used for primary data storage and plotting some graphs, respectively.

### Culture profile and CQAs of mAb in basal medium

**Fig. 2:**
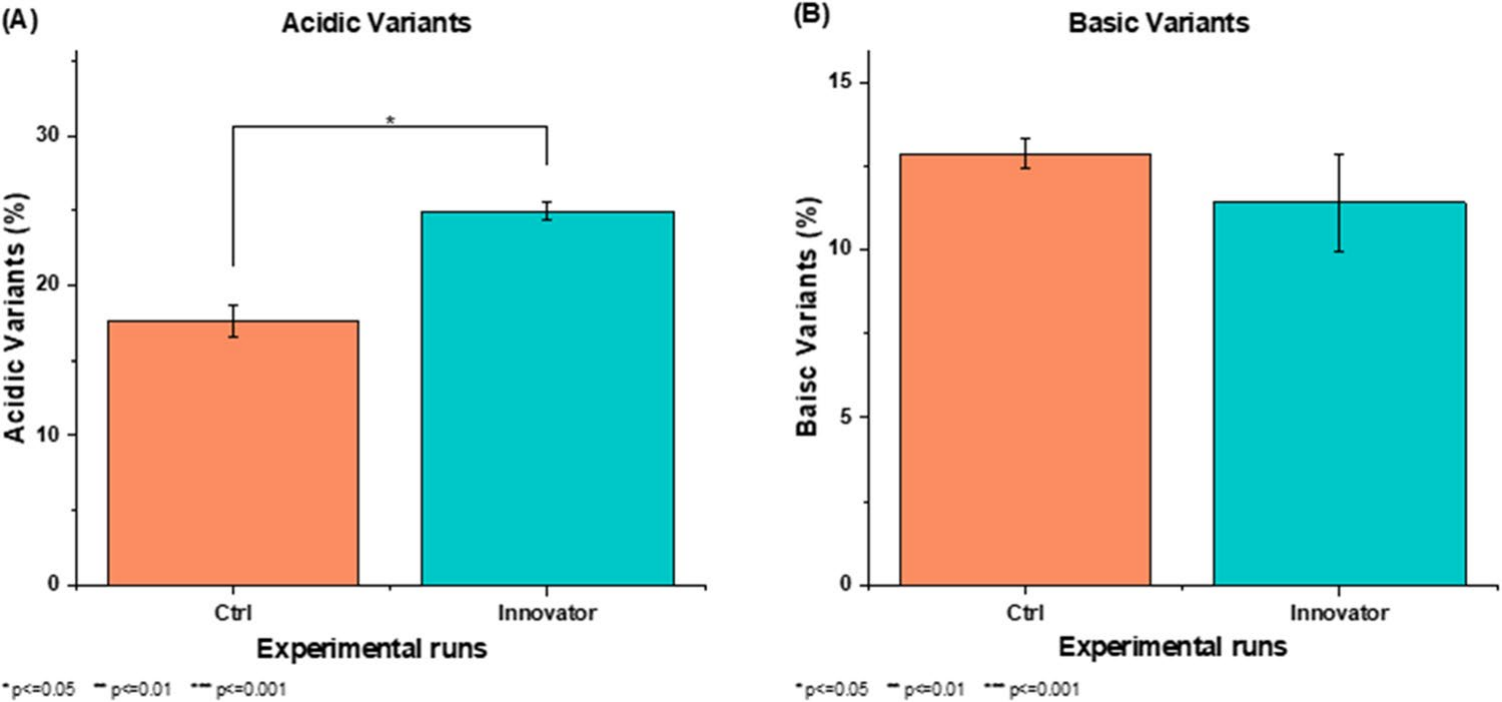
Comparison of charge variant profile of (**A**) acidic and (**B**) basic variants with respect to innovator molecule (*N* = 2) (*p = 0.05, **p = 0.01, ***p = 0.001)

The CHO cell line was grown in suspension culture in a shaker flask in fed-batch mode to evaluate the charge variant profile of the basal (control) medium. The initial cell concentration was determined at (0.5 ± 0.05) × 10^6^ cells/mL. Shaker flask (SF-125) cultures were run in duplicates in the fed-batch mode for 10 days, with 10% of efficient feed supplementation on alternate days from 3rd day to 9th day. The charge variant profile of mAb was evaluated after harvest collection at the end of culture, i.e., harvest at day 10 and acidic (17.64 ± 1.07)% and basic variants (12.86 ± 0.43)% were estimated. The charge variant profile was compared to that of the innovator molecule, acidic (24.97 ± 0.54)% (Fig. 2A) and basic (11.41 ± 1.44)% variants (Fig. 2B). Acidic variant composition were found to be significantly lower (*p* < 0.02, unpaired *t*-test assuming equal variance) in the basal medium when compared to the innovator (Herceptin®). The basic variant differences were relatively non-significant when compared to the innovator product (*p* > 0.3, unpaired *t*-test assuming equal variance).

Next, the impact of metal ions (Cu, Fe, Zn, Mn, Mg, Co, and Ni) on charge variant profile of the resulting product was examined. Various media formulations were generated as described in Section dataset of material and methods and were subjected to culture in batch mode to evaluate the effect of supplements on charge variants. Formulation composition and the corresponding charge variant profile have been provided (Table S1).

### Feature ranking

**Fig. 3:**
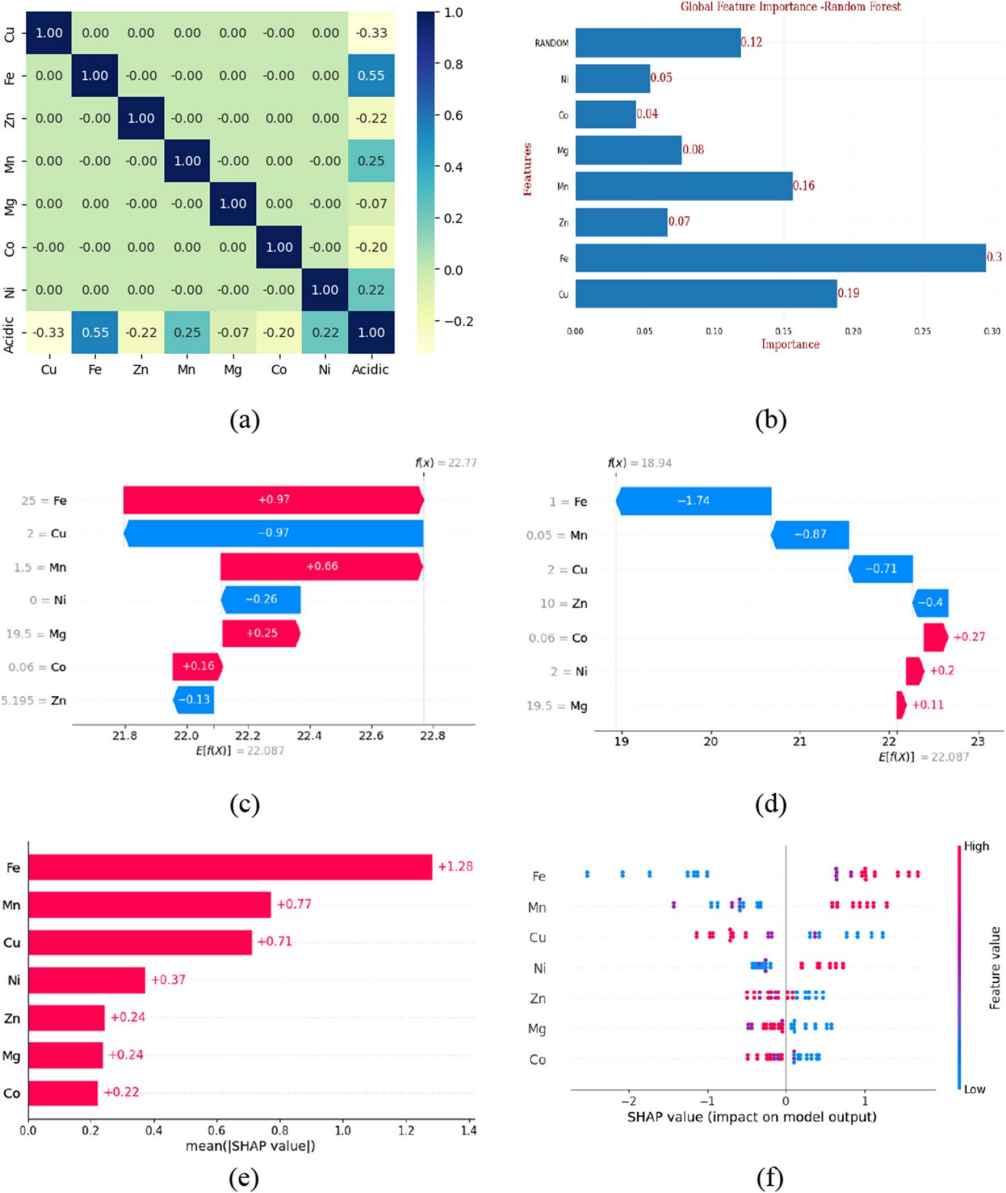
Feature ranking for acidic variants (*N* = 2). **a** Pearson’s correlation coefficient (PCC), **b** Gini feature ranking, **c** waterfall plot (random observation 1), **d** waterfall plot (random observation 2), **e** absolute mean SHAP value, and **f** bee swarm plot

**Fig. 4:**
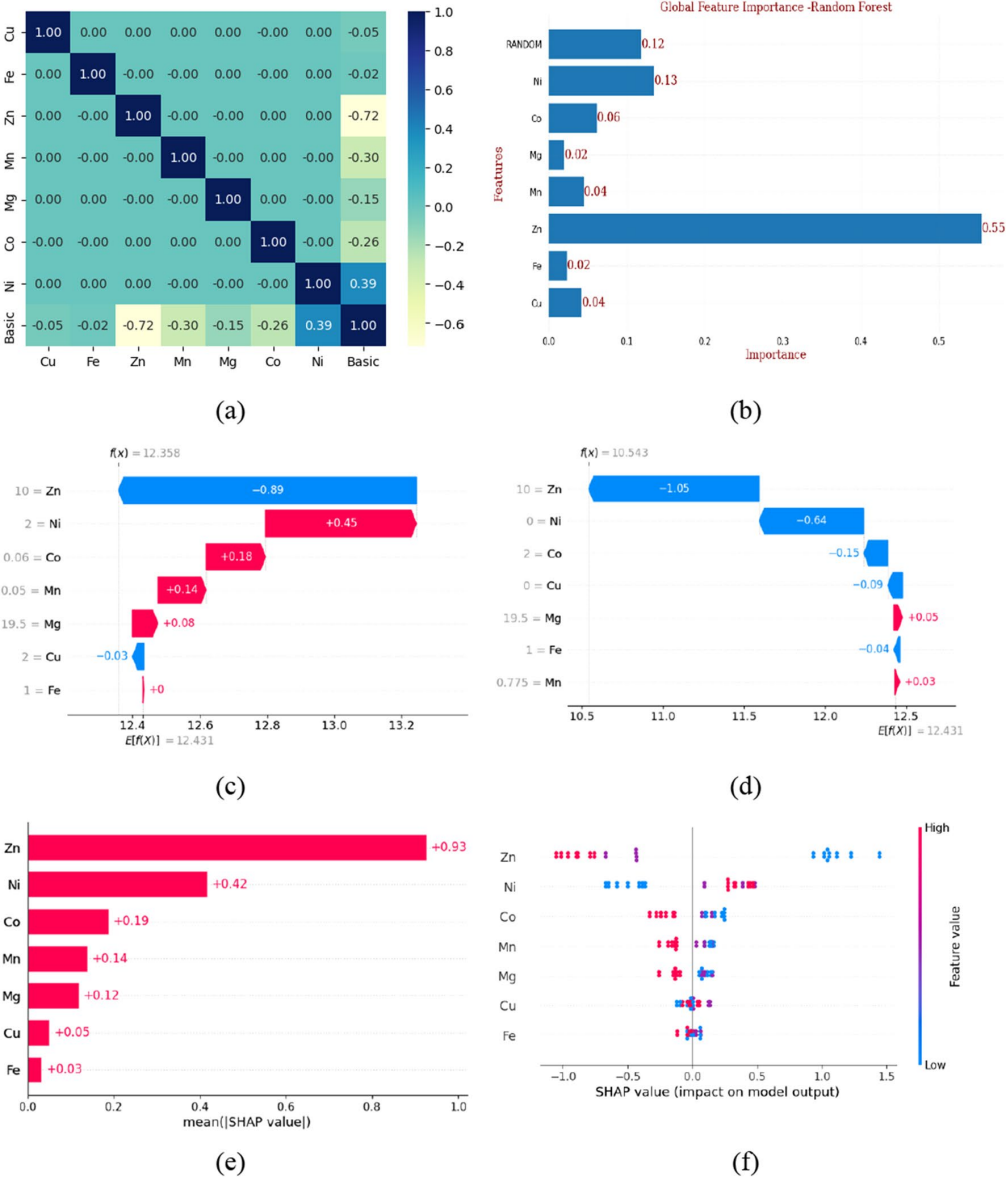
Feature selection basic variants (*N* = 2). **a** Pearson’s correlation coefficient (PCC), **b** Gini feature ranking, **c** waterfall plot (random observation 1), **d** Waterfall plot (random observation 2), **e** absolute mean SHAP value, and **f** bee swarm plot

Cell culture was performed in 34 medium formulations, and the temporal changes in cell culture were measured at 24-h or 48-h intervals in duplicates (*N* = 2). For feature ranking, both traditional univariate biostatistics analyses followed by a ML analysis were employed. Figures 3 and 4 illustrate the outcomes for feature ranking of the acidic and basic charge variations, respectively.

Filter-based technique, i.e., Pearson’s correlation coefficient (or Pearson product-moment correlation coefficient, PCC), indicates a linear correlation between elements of two lists. The absolute value of PCC produces a high value (close to 1) if linear correlation is present, and a low value (close to 0) if not (Sedgwick 2012). Based on the correlation matrix, Fe (PCC =  + 0.55) exhibited maximum positive correlation with acidic variants followed by Cu and Mn. While copper is associated negatively with acidic variants, Mn is positively correlated but to a lesser extent (Fig. 3a). In the case of basic variants, Zn (PCC =  − 0.72) exhibited maximum and negative correlation (Fig. 4a), followed by Ni (Fig. 4a).

For ML feature ranking, we focused on embedded-based methods for feature selection. Random forests (RF) and boosting techniques like gradient boost decision trees (GBDT) were used (Fig. S3). The “Gini method” was also evaluated with the addition of a random variable, feature scores above the score of random features were considered significant and taken into further consideration. In the case of acidic variants, Fe, Mn, and Cu were the best performers with scores of 0.3, 0.19, and 0.16, respectively, which are above the score of random/dummy features, i.e., 0.12 (Fig. 3b). In the case of basic variants, Zn and Ni exhibit a prominent role (Fig. 4).

SHapley Additive exPlanations (SHAP) is a game theoretic approach to explain the output of any ML model. The basic Shapley values from game theory and their related extension are used to correlate optimal credit allocation with local feature explanations (Lundberg and Lee 2017; Lundberg et al. 2018, 2020; Mitchell et al. 2022). Waterfall plots (Figs. 3c, d and 4c, d) are designed to display explanations for individual predictions, so they expect a single row of an explanation object (single observation) as input. The bottom of a waterfall plot starts at the expected value of the model output, and then, each row shows how the positive (red) or negative (blue) contribution of each feature moves the value from the expected model output over the background dataset to the model output for this prediction. Waterfall plot (Fig. 3c) SHAP explains the random forest, regressor model in terms of expected model outcome, i.e., *E*[*f*(*x*)] = 22.087 is the average predicted outcome for the model across all the observations. The *Y*-axis represents the actual feature value for this observation. *f*(*x*) = 18.94 represents the outcome for this specific observation. SHAP values (blue and red arrows) represent the contribution of each component to the outcome of the respective observation, i.e., *f*(*x*) with respect to the average. In this observation, Fe at the concentration 25 ppm contributes positively (+ 0.97 units) to increasing acidic variants while Cu at conc 2 ppm decreases acidic variants (− 0.97 units). In another observation (Fig. 3d) where Fe supplementation is at lower end (1 ppm) acidic variants reduces (− 1.74 units) with respect to expected value, i.e., *E*[*f*(*x*)] = 22.087.

The absolute mean SHAP value (Figs. 3e and 4e) is the sum of all SHAP values for a particular feature across all the observations. Hence, features having max value contribute most significantly to the model. In this study, Fe exhibits the most significant impact on acidic variants (Fig. 3e) and Zn on basic variants (Fig. 4e). The bees warm plot (Fig. 3f) represents the plot of all individual SHAP values, illustrating not only the extent of contribution of each feature but also the positive or negative impact on the model outcome. For acidic variants, positive correlation with Fe and Mn concentrations and negative correlation with Cu concentration are observed (Fig. 3f). For basic variants, negative correlation with Zn concentration is observed (Fig. 4f).

**Fig. 5:**
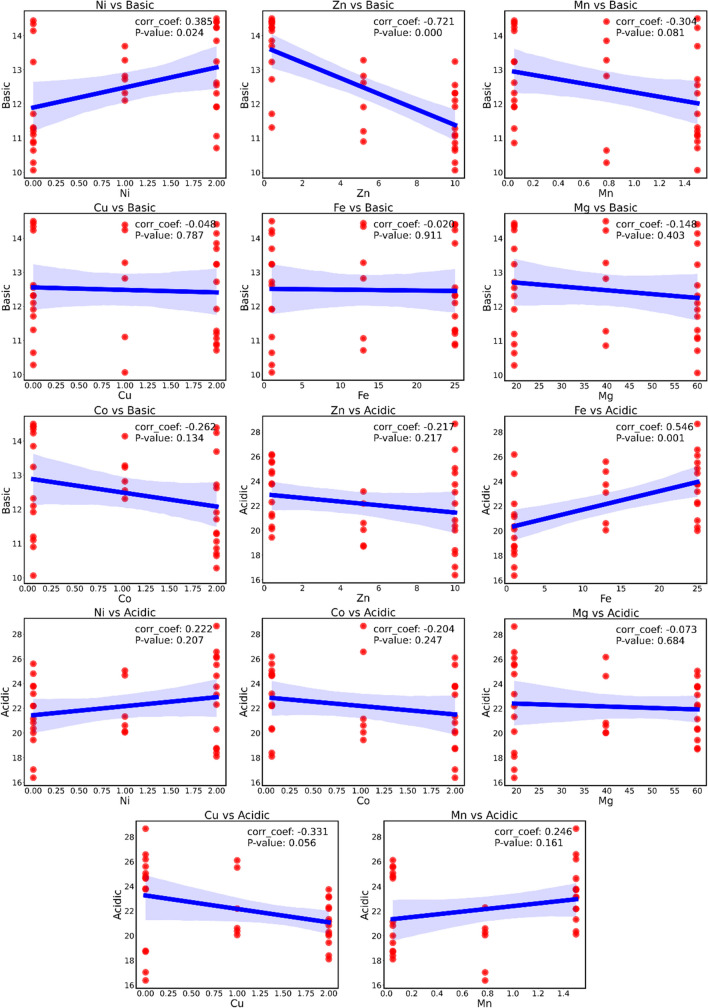
Spearman correlation scatter plots with linear regression (blue line) with its confidence interval (blue area) for both acidic and basic charge variants with correlation coefficient (corr_coef) and *p*-value

When the *p*-value is less than 0.05, it indicates that the null hypothesis is not significant, which leads to the rejection of the null hypothesis. On the other hand, when the *p*-value is more than 0.05, the null hypothesis is maintained, and a *p*-value of 0.01 provides more compelling evidence for the rejection of the null hypothesis. From Fig. 5, it is evident that Fe (corr_coef = 0.546 and *p*-value = 0.001) demonstrates substantial correlation and is statistically significant, indicating a robust relationship for acidic charge variant. Conversely, Zn (corr_coef =  − 0.217 and *p*-value = 0.217), Ni (corr_coef = 0.222 and *p*-value = 0.207), Co (corr_coef =  − 0.204 and *p*-value = 0.247), Mg (corr_coef =  − 0.073 and *p*-value = 0.68), Mn (corr_coef = 0.246 and *p*-value = 0.161) exhibits correlation with varying strengths, but none of these relationships reach statistical significance for acidic charge variant. Cu (corr_coef =  − 0.331 and *p*-value = 0.056) is suggesting a moderate negative correlation, while its *p*-value of 0.056 approaches significance but does not meet the conventional threshold and purpose of the objective. Similarly, for the basic charge variant, Zn (corr_coef =  − 0.721 and *p*-value = 0.000) and Fe (corr_coef = 0.385 and *p*-value = 0.024) exhibit statistical significant.

### Prediction of charge variants using featured variables

Fe with Pearson’s correlation coefficient (PCC =  + 0.55) and Zn (PCC =  − 0.72) were further used as the potent modulators for acidic and basic variants, respectively. Several culture flasks were run with varying concentrations of Fe and Zn to make an ML predictive model for the charge variant profile. A total of 42 experimental runs having different combinations of Fe and Zn were generated. To evaluate the robustness and generalizability of the proposed framework, we utilized these 42 data for testing purposes. Fig. S4 depicts the acidic and basic variants with respect to Fe and Zn, respectively.

Starting with the whole dataset *D* (Table S2), we generated a collection *D* = ${\{\left\{{D}_{i}^{tr}, {D}_{i}^{ts}\right\}\}}_{i=1}^{N}$ of *N* randomly generated training/test partitions *D* = ${D}_{i}^{tr}\bigcup {D}_{i}^{ts}$ with test fraction of 0.2 wrt to whole dataset. On the *N* training portions, ${\left\{{D}_{i}^{tr}\right\}}_{i=1}^{N}$, we employed several supervised learning algorithms to predict the charge variants, and their performances are summarized in Table 2. Tree-based regression models, i.e., random forest (Breiman 2001), decision tree (Podgorelec et al. 2002; Kotsiantis 2013), extra tree regressor (Martiello Mastelini et al. 2023; Basu 2020), and boosting-based algorithms such as CatBoost regressor (Hancock and Khoshgoftaar 2020), Xgboost, i.e., extreme gradient boosting (Babajide Mustapha and Saeed 2016), gradient boosting regressor (Li et al. 2018) outperformed the other regressors. While linear models with regularization (lasso, ridge) (Ranstam and Cook 2016; Zou and Hastie 2005), elastic net (Zou and Hastie 2005), and support vector regressors (Smola et al. 2004) exhibited moderate performance. A summary of performance metrics (*R*^2^, Adj-*R*^2^) for all evaluated models is provided in Table 2. All model scores were cross validated with validation set splitting (cv = 6), and random shuffle, the mean *R*^2^, and its standard deviation were evaluated to get the spread of the coefficient of determination.

**Table 2:** Summary of evaluation matrices of regression model: mean absolute error (MAE), mean squared error (MSE), root mean squared error (RMSE), coefficient of determination (*R*^2^), Adj-*R*^2^, mean *R*^2^ after cross-validation, and standard deviation of *R*^2^

Model	MSE	RMSE	*R*^2^	${{\text{Adj}}\_R}^{2}$	Mean *R*^2^	Stdv *R*^2^
XGBoost	0.88	0.94	0.93	0.92	0.9199	0.0410
GBR	0.87	0.93	0.93	0.92	0.9288	0.0441
DT	0.88	0.94	0.93	0.92	0.9252	0.0450
CatBoost regressor	0.88	0.94	0.93	0.92	0.8861	0.0594
SVR	0.86	0.93	0.93	0.92	0.7664	0.1643
ET	1.0	1.0	0.92	0.91	0.9057	0.0842
Elastic net	0.97	0.99	0.92	0.91	0.8343	0.0854
RF	0.91	0.95	0.92	0.91	0.9151	0.0455
BRR	1.08	1.04	0.91	0.9	0.8239	0.1045
LLAR	1.12	1.06	0.91	0.9	0.8204	0.1092
Lasso	1.08	1.04	0.91	0.9	0.8231	0.1041
Ridge regression	1.12	1.06	0.91	0.9	0.8205	0.1091
LR	1.12	1.06	0.91	0.9	0.8204	0.1092
KNN	1.24	1.11	0.89	0.88	0.8036	0.3132
Huber	1.43	1.19	0.88	0.87	0.8441	0.0768

**Fig. 6:**
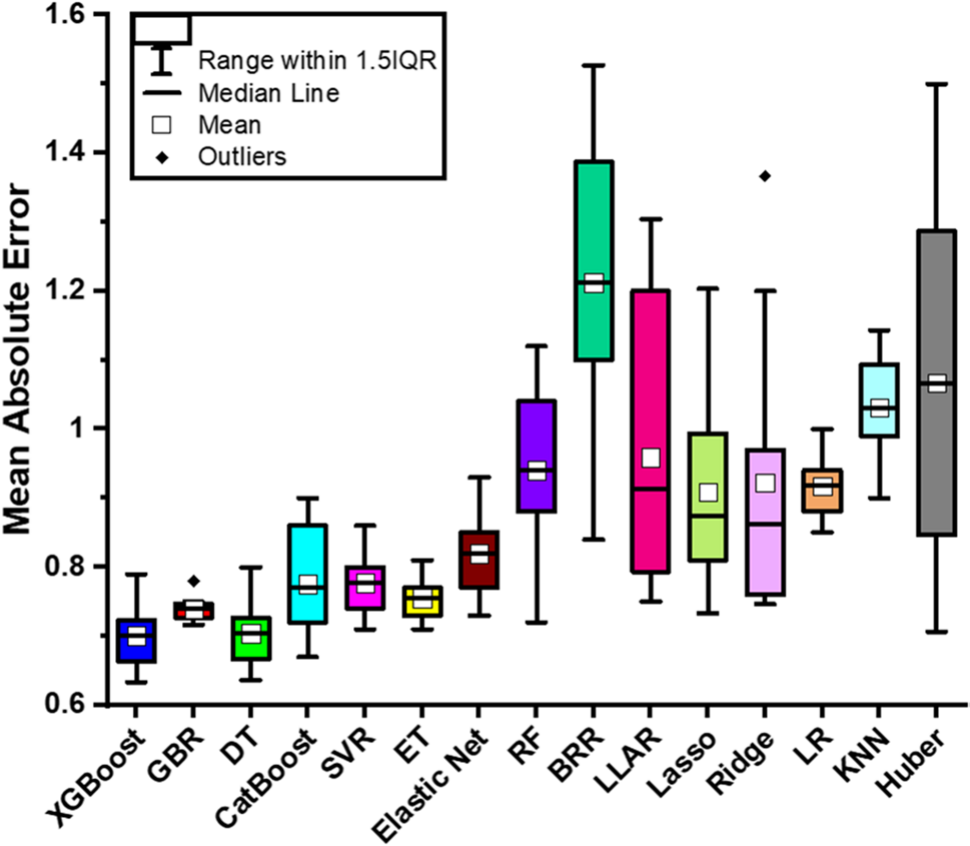
Box plots comparing the performance of different machine learning techniques in terms of mean absolute error

**Fig. 7:**
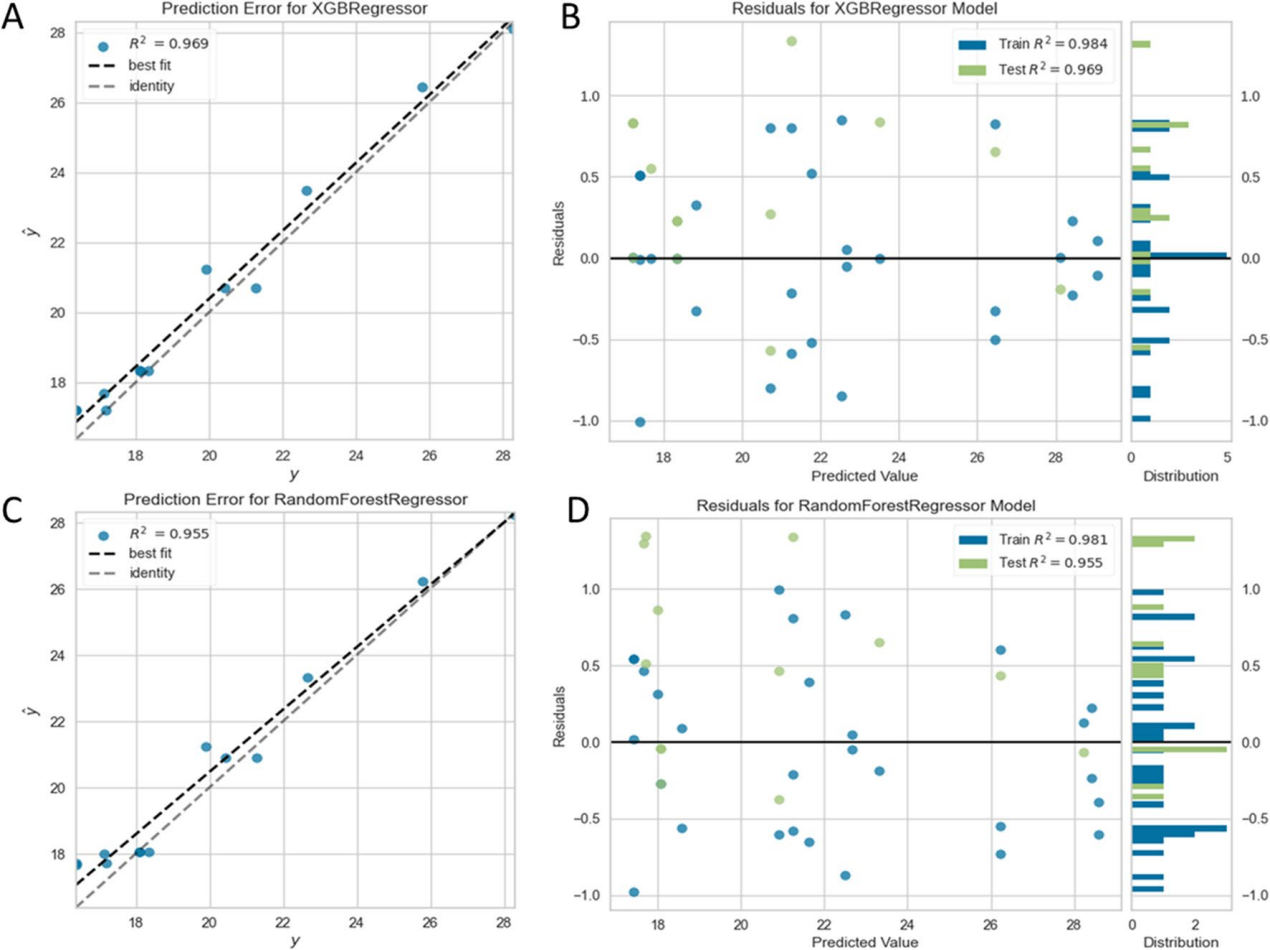
Prediction with extreme gradient boost regressor: (**A**) observed (*y*) vs. predicted (*ŷ*) (error) plot; (**B**) residual plot, prediction with random forest; (**C**) observed (*y*) vs predicted (*ŷ*) (error) plot; (**D**) residual plot

Figure 6 illustrates the box plots of fifteen different machine learning techniques in terms of mean absolute error. For each machine learning approach, these values indicate the average absolute difference between the values that were predicted and the actually obtained. Lower MAE values are indicative of greater performance as they suggest that the model’s predictions are more consistent with the actual values. Based on the MAE values that have been provided, it is observed that XGBoost, GBR, and DT have the lowest MAE value, which indicates that their performance is considerably superior to that of other strategies. Huber regression has the highest MAE scores, which indicates that the performance is comparatively low. Most of the models used the “scikit-learn” library with their respective modules except the boosting techniques (Xgboost, catboot, etc.) which have their specific libraries. We evaluated models with both feature scaling and without feature scaling, depending on the requirements of the models. Observed vs. predicted (error analysis) plot and residual plot are shown in Fig. 7A, B using the random forest as a regressor (*R*^2^ test = 0.955).

In Fig. 7A–D, a comparison of the prediction error and residuals for two different models (XGBoost and random forest regressor) fitted on our dataset. XGBoost model shows slightly higher values of *R*^2^ scores compared to the random forest model. It can be inferred that the XGBoost regression has less residual errors than random forest model. Random forest (*R*^2^: 0.9151 ± 0.0455) and gradient boost regressor (*R*^2^: 0.9288 ± 0.0441) were among the top performers based on the mean coefficient of determination (*R*^2^) scores.

### Optimization of cell culture medium

From the ML model constructed with the initial training dataset, approximately 625 simulated medium candidate formulations were obtained by altering the concentrations of the medium components across numerous variations. By inputting the 625 media candidates into the ML model, the relative cell culture, represented by charge variants, was predicted. The gradient boosting decision tree (GBDT) model was used to predict the medium combinations leading to a required charge variant, i.e., equivalence to the innovator molecule. Hyperparameter tuning was performed as per the parameters described in machine learning methods subsection media optimization using both grid and randomized search. Both approaches delivered comparable results, but grid search is computationally intensive and takes a longer time in comparison to randomized search.

The medium combinations that correspond to the required target of the innovator charge variant profile, i.e., (24.97 ± 0.54)% acidic and (11.41 ± 1.44)% basic variants, were screened. Simulated media candidates predicting charge variant composition outside these ranges were filtered out. Based on the model prediction, Fe concentration between 10 and 25 ppm and Zn concentration between 5.5 and 12.5 ppm was estimated. Experimental validation was performed with 20 ppm of Fe and 5.5 ppm of Zn as existing data suggest that higher concentrations do not significantly impact charge variant composition. The control flask and optimized medium cell culture flask were cultured in fed-batch mode as described in the cell culture subsection of the material and methods section.

**Fig. 8:**
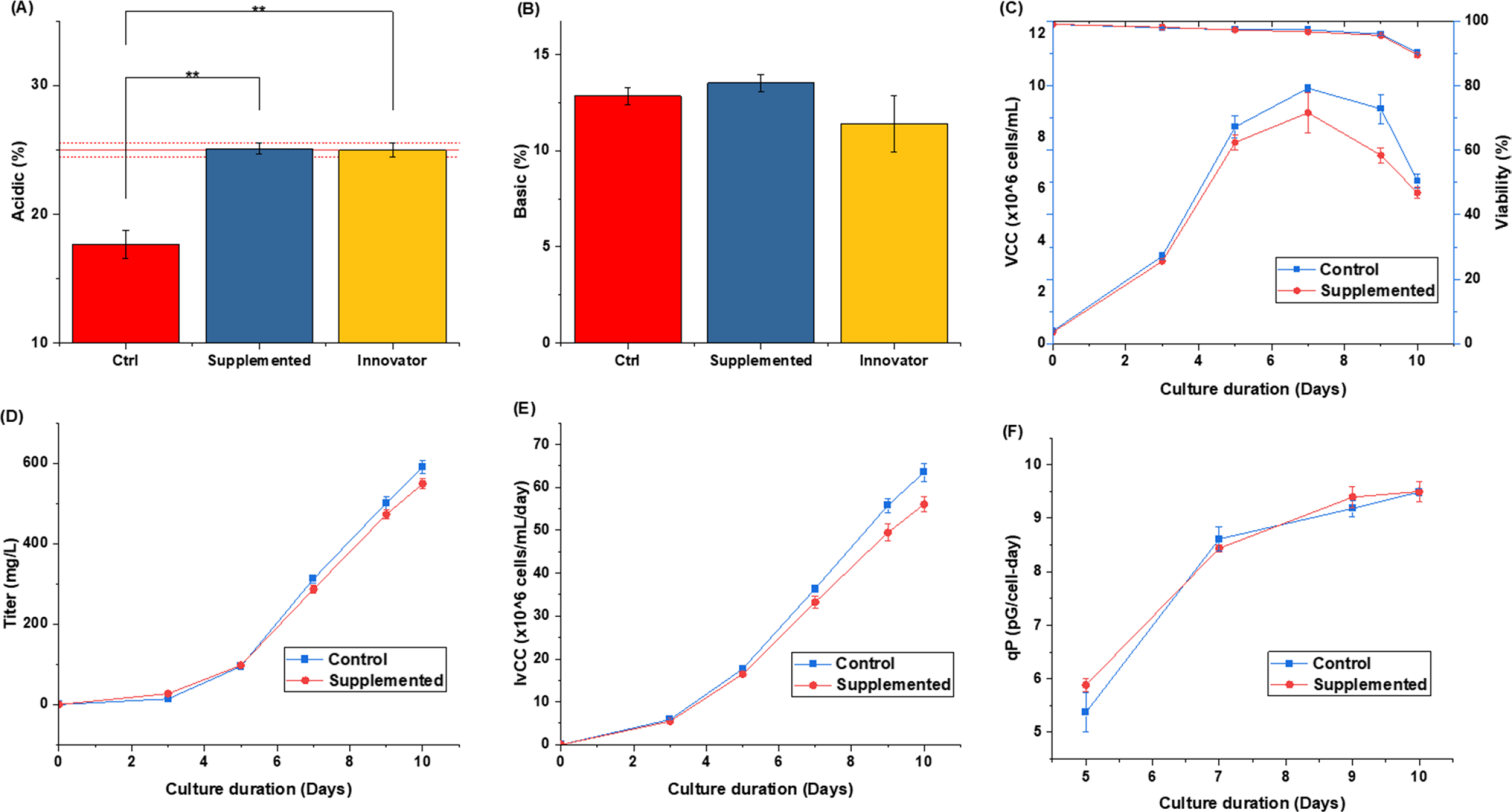
Optimized medium cell culture and charge variant profile. **A** Acidic variants (%). **B** Basic variants (%). **C** Viability (upper) and VCC (lower). **D** Titer (mg/L). **E** Integral of viable cell density (IVCC). **F** Specific productivity (qP) (*N* = 2) (*p = 0.05, **p = 0.01, ***p = 0.001)

Effects of supplements on culture profile and antibody production were evaluated by comparing the charge variant profiles for the control and treated shaker flasks with innovator (Fig. 8 and Table S3). Overall viability in both cases was similar but the viable cell concentration (VCC) was higher in control compared to treated with peak VCC for control (~ 10 × 10^6^ cell/mL) and treated (~ 8.9 × 10^6^ cell/mL) (Fig. 8C). The same was reflected in IvCC (Fig. 8E), and hence, decreased titer (~ 7%) (Fig. 8D) was also observed in the treated culture, which may be attributed to an increase in the oxidative environment (Handlogten et al. 2018). The slight decrease in titer is mainly attributed to decrease in VCC as specific productivity (qP) (Fig. 8F) was similar in both the cases.

## Discussion

Developing and optimizing cell culture media is a tedious and time-consuming process, compared to normal biochemical reactions due to the complex interaction of cells with media components. Media components are known to modulate critical quality attributes (CQAs) like glycosylation and charge variants (Xie et al. 2016; Rathore et al. 2017; Miao et al. 2017; Gangwar et al. 2021, 2022; Romanova et al. 2022; Zhang et al. 2023). In this study, we have demonstrated the application of an ML-based approach involving feature selection, prediction, and optimization to identify metal ions and their concentrations for achieving charge variant composition of the innovator product. Compared to our previous studies (Gangwar et al. 2021, 2022) regarding the optimization of metal ions using design of experiments (DOE), machine learning-based optimization serves some additional advantages. These include better prediction capabilities of ML models (*R*^2^ = 0.95) compared to conventional statistical analysis (*R*^2^ = 0.85). DOE analysis often restricts the concentration range of evaluating components as per the design matrix, while in ML model training, there are no such restrictions. This is quite helpful in the case of cell culture media component evaluation where freedom of manipulation of the component’s concentration is often restricted because of many limitations. DOE has another constraint related to the number of subjects that can be screened, evaluated, and optimized while ML can handle hundreds of variables smoothly.

The charge variant profile of in-house produced mAb was quite different from the innovator molecule with the acidic and basic variant compositions in the innovator product (Herceptin®) of 24.97 ± 0.54% and 11.41 ± 1.44% and for the in-house product 17.64 ± 1.07% and 12.86 ± 0.43%, respectively. Seven metal ions (Cu, Fe, Zn, Mn, Mg, Co, and Ni) used in the screening study were analyzed and shortlisted based on ML-based feature selection approaches. In the case of acidic variants, Fe, Mn, and Cu exhibited the most significant impact with Fe and Mn promoting the formation of acidic variants and Cu inhibiting the formation of the acidic variants. For basic variants, Zn and Ni have the most significant impact with Ni exhibiting a positive and Zn negative correlation with % basic variants. The optimized concentration of Fe and Zn as per the gradient boost decision tree (GBDT) algorithm was estimated as 20 ppm and 5.5 ppm, respectively. With the optimized concentration of Fe and Zn, we were able to get charge variant profiles with acidic variants (25.1 ± 0.31)% and basic variants (13.5 ± 0.30)%. The charge variant profile was quite close to the innovator molecule with no significant difference, acidic (*p* = 0.815, two-sided *t*-test assuming equal variance) and basic variants (*p* = 0.185, two-sided *t*-test assuming equal variance) concerning the innovator molecule.

Fe is one of the crucial media components required for the growth and proper functioning of cells. Various hemoproteins and nonheme proteins depend on Fe for their proper functioning, which are involved in oxygen metabolism (oxidase, peroxidase, catalase, etc.), key reactions of energy metabolism mitochondrial aconitase, and [Fe-S] proteins of electron transport system (Ponka 1999). Its optimum concentration is important not only to get a favorable viable cell count (peak VCC) but also for protein production (Xu et al. 2018). Excess Fe has been linked to deleterious effects because of the formation of reactive oxygen species (ROS) which can cause damage to cells (Ponka 1999). Indeed, higher Fe concentrations beyond a point result in reduced titer (Fig. 8D), primarily due to a decrease in the overall integral of viable cell count (IVCC) but without significant change in specific productivity of cell (qp) (Fig. 8F). Other quality attributes like aggregation did not seem to be significantly impacted in the experiments performed (Fig. S2).

Zn is also an important element in cytoprotectant processes and regulates energy metabolism (Yang et al. 2017). It is an important regulator of caspase-dependent apoptosis of cells and its optimum amount in the medium is suggested to suppress apoptosis. Again, more than the optimum concentration of Zn can induce cell death either by apoptosis or necrosis (Truong-Tran et al. 2001). Zn deficiency in media may also depress G1/S cell cycle progression in certain cell types (Wong et al. 2007).

Screening and optimization of media components have a significant impact on process economics and for a biosimilar manufacturer, on biosimilarity of the resulting product. While conventional methods are used regularly, the ML approach offers a significantly more efficient screening of media components. In this study, we explore the applicability of ML for screening of metal ions for their effect on the charge variant profile, identification of the metal ions that impact the most and estimating their optimal concentrations. Feature ranking using random forest shortlisted iron as a candidate for modulating acidic variant composition and Zn for basic variant composition. While small variations in the cell culture profile were observed in terms of viable cell density and titer, there were hardly any variations in terms of specific productivity (qP), and no aggregation was observed in any culture control or supplemented. The proposed approach would be of interest to those working on the production of biosimilar products or innovators looking for an end-to-end ML approach from media component screening and predicting CQAs to optimization.

However, the proposed study exhibits strengths in machine learning applications but also encounters limitations such as dataset specificity, implementation challenges, and decision-making processes. To overcome these challenges, our study focused on conducting a thorough examination and strategic feature selection, then assessing the models using various classification approaches. Acknowledging the current limitation of evaluating a relatively small dataset, it is essential that validation efforts be expanded to significantly larger. To improve scalability and robustness, especially on bigger datasets, we prioritize hyperparameter tuning and optimization algorithm exploration, fine-tuning learning rate, tree depth, and regularization. In the future, we aim to explore the integration of active learning to improve the flexibility of our models, offering the potential to enhanced adaptability and efficiency in training.

## Supplementary Information

Below is the link to the electronic supplementary material.

Supplementary file1 (PDF 308 KB)

## Data Availability

The data that support the findings of this study are available in the supplementary material.
