# Supplementary material for: Explainable AI for CHO cell culture media optimization and prediction of critical quality attribute
Source: Appl Microbiol Biotechnol. 2024 Apr 24;108(1):308. doi: 10.1007/s00253-024-13147-w (PMC11043154; doi:10.1007/s00253-024-13147-w)
Supplement: Supplementary file 1 — Supplementary file1 (PDF 308 KB) [file 253_2024_13147_MOESM1_ESM.pdf]

Applied Microbiology and Biotechnology

Leveraging Explainable AI for CHO Cell Culture Media Optimization and Prediction of Critical Quality Attribute

Neelesh Gangwar[1], Keerthiveena Balraj[2], Anurag S. Rathore[2,3*]

[1]School of Interdisciplinary Research, Indian Institute of Technology Delhi, New Delhi, 110016, India
[2]Yardi School of Artificial Intelligence, Indian Institute of Technology Delhi, New Delhi, 110016, India
[3]Department of Chemical Engineering, Indian Institute of Technology Delhi, New Delhi, 110016, India

Corresponding to:
Anurag Singh Rathore, Professor, Department of Chemical Engineering, Joint Faculty, Yardi School of Artificial Intelligence, Indian Institute of Technology Delhi, Hauz Khas, New Delhi, 110016, India, Phone +91-9650770650, Email: asrathore@biotechcmz.com

Supplementary data

Table S1: A Total of seven metal ions were taken as features or predictors, while a total of 34 formulations including duplicates (N=2) used in the experiment. Hence a tabular dataset (D_{34x9}) having 34 rows (observations), and 9 columns (Features) which include 2 target variables was ready for feature ranking and selection.

SampleID	Cu	Fe	Zn	Mn	Mg	Co	Ni	Acidic (%)	Basic (%)
P3S1	2.00	1.00	10.00	0.05	19.50	0.06	2.00	18.10	13.24
P3S2	0.00	1.00	5.20	0.05	60.00	2.00	2.00	18.70	11.91
P3S3	2.00	1.00	0.39	0.05	60.00	1.03	0.00	21.14	14.14
P3S4	0.00	1.00	10.00	0.78	19.50	2.00	0.00	17.03	10.64
P3S5	2.00	1.00	0.39	1.50	19.50	2.00	1.00	20.13	12.72
P3S6	0.00	1.00	0.39	1.50	39.75	0.06	2.00	24.63	14.50
P3S7	1.00	1.00	10.00	1.50	60.00	0.06	0.00	20.36	11.10
P3S8	0.00	13.00	0.39	0.05	19.50	0.06	0.00	24.80	14.34
P3S9	1.00	13.00	5.20	0.78	39.75	1.03	1.00	20.05	13.28
P3S10	2.00	13.00	10.00	1.50	60.00	2.00	2.00	23.10	10.71
P3S11	1.00	25.00	0.39	0.05	19.50	2.00	2.00	26.10	14.24
P3S12	2.00	25.00	10.00	0.05	39.75	2.00	0.00	20.01	11.28
P3S13	0.00	25.00	10.00	0.05	60.00	0.06	1.00	25.05	12.32
P3S14	2.00	25.00	0.39	0.78	60.00	0.06	2.00	22.30	13.85
P3S15	0.00	25.00	10.00	1.50	19.50	1.03	2.00	26.57	12.55
P3S16	2.00	25.00	5.20	1.50	19.50	0.06	0.00	23.17	10.90
P3S17	0.00	25.00	0.39	1.50	60.00	2.00	0.00	23.75	11.31
P3S18	2.00	1.00	10.00	0.05	19.50	0.06	2.00	18.39	11.92
P3S19	0.00	1.00	5.20	0.05	60.00	2.00	2.00	18.77	12.62
P3S20	2.00	1.00	0.39	0.05	60.00	1.03	0.00	19.43	13.23
P3S21	0.00	1.00	10.00	0.78	19.50	2.00	0.00	16.37	10.28
P3S22	2.00	1.00	0.39	1.50	19.50	2.00	1.00	21.33	13.69
P3S23	0.00	1.00	0.39	1.50	39.75	0.06	2.00	26.18	14.23
P3S24	1.00	1.00	10.00	1.50	60.00	0.06	0.00	22.19	10.06
P3S25	0.00	13.00	0.39	0.05	19.50	0.06	0.00	25.60	14.44
P3S26	1.00	13.00	5.20	0.78	39.75	1.03	1.00	20.60	12.82
P3S27	2.00	13.00	10.00	1.50	60.00	2.00	2.00	23.74	11.06
P3S28	1.00	25.00	0.39	0.05	19.50	2.00	2.00	25.52	14.39
P3S29	2.00	25.00	10.00	0.05	39.75	2.00	0.00	20.83	10.86
P3S30	0.00	25.00	10.00	0.05	60.00	0.06	1.00	24.67	12.10
P3S31	2.00	25.00	0.39	0.78	60.00	0.06	2.00	20.29	14.41
P3S32	0.00	25.00	10.00	1.50	19.50	1.03	2.00	28.66	12.31
P3S33	2.00	25.00	5.20	1.50	19.50	0.06	0.00	22.19	11.20
P3S34	0.00	25.00	0.39	1.50	60.00	2.00	0.00	23.81	11.71

Table S2: A total of 42 media candidates have been tested for generating the complete data set required to learn the supervised regression models.

SampleID	Fe	Zn	Acidic	Basic
P3S35	1	0.4	16.88	13.165
P3S36	7	0.4	19.91	12.93
P3S37	13	0.4	26.96	14.23
P3S38	25	0.4	28.66	13.47
P3S39	1	4.7	19.15	12.64
P3S40	1	8.4	17.2	10.56
P3S41	1	12	18.34	10.82
P3S42	1	0.4	16.88	11.165
P3S43	7	0.4	21.46	12.32
P3S44	13	0.4	26.78	14.5
P3S45	1	3.3	17.13	10.31
P3S46	7	3.3	20.44	12.05
P3S47	7	3.3	21.28	12.62
P3S48	13	3.3	22.62	12.14
P3S49	1	6.2	17.2	10.56
P3S50	7	6.2	21.24	11.65
P3S51	13	6.2	23.37	12.37
P3S52	25	6.2	29.18	15.86
P3S53	1	12	18.34	10.82
P3S54	13	12	23.5	13.36
P3S55	25	12	28.3	13.99
P3S56	1	0.4	17.4	12.55
P3S57	7	0.4	20.44	11.67
P3S58	13	0.4	25.8	13.94
P3S59	25	0.4	28.2	13.47
P3S60	1	4.7	18.5	12.23
P3S61	1	8.4	16.37	10.06
P3S62	1	12	18.11	10.94
P3S63	1	0.4	18.4	12.55
P3S64	7	0.4	21.83	10.69
P3S65	13	0.4	25.63	12.85
P3S66	1	3.3	17.68	10.27
P3S67	7	3.3	19.91	12.93
P3S68	7	3.3	21.51	11.52
P3S69	13	3.3	22.72	12.96
P3S70	1	6.2	16.37	10.06

P3S71	7	6.2	22.28	11.54
P3S72	13	6.2	21.67	12.4
P3S73	25	6.2	28.97	15.86
P3S74	1	12	18.11	10.94
P3S75	13	12	22.66	14.26
P3S76	25	12	28.11	15.96

Table S3: Charge variants estimation of control and validation in fed-batch mode

	**Acidic**	**Baisc**
**Ctrl**	16.88	13.165
**Ctrl**	18.4	12.55
**Supplemented**	24.79	13.22
**Supplemented**	25.41	13.83
**Innovator**	25.35	12.43
**Innovator**	24.59	10.39

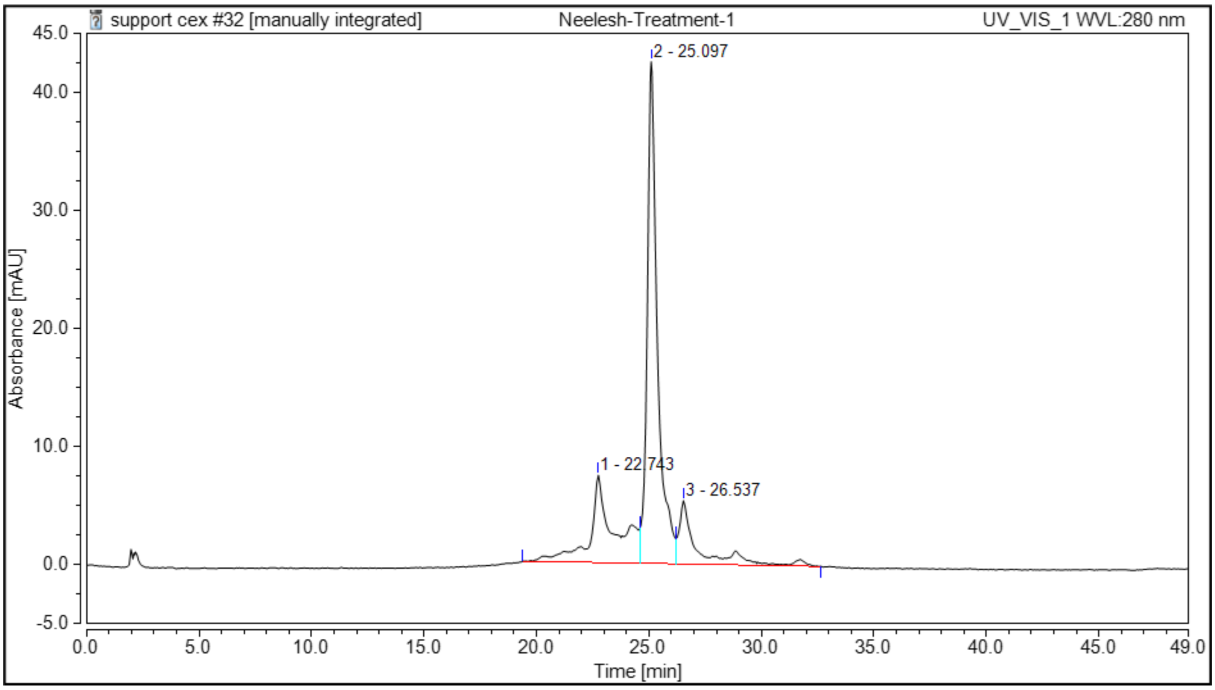

Fig. S1 CEX Chromatogram of supplemented culture

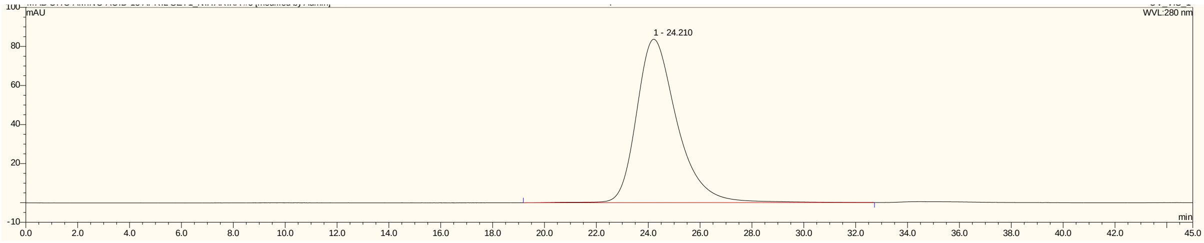

Fig. S2: Size Exclusion chromatography (SEX) profile

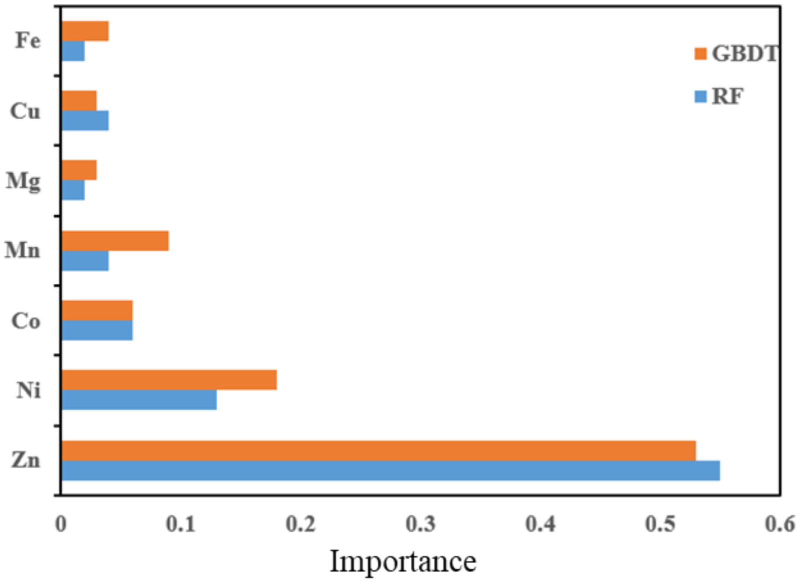

Figure S3 Comparison between RF and GBDT in terms of feature ranking.

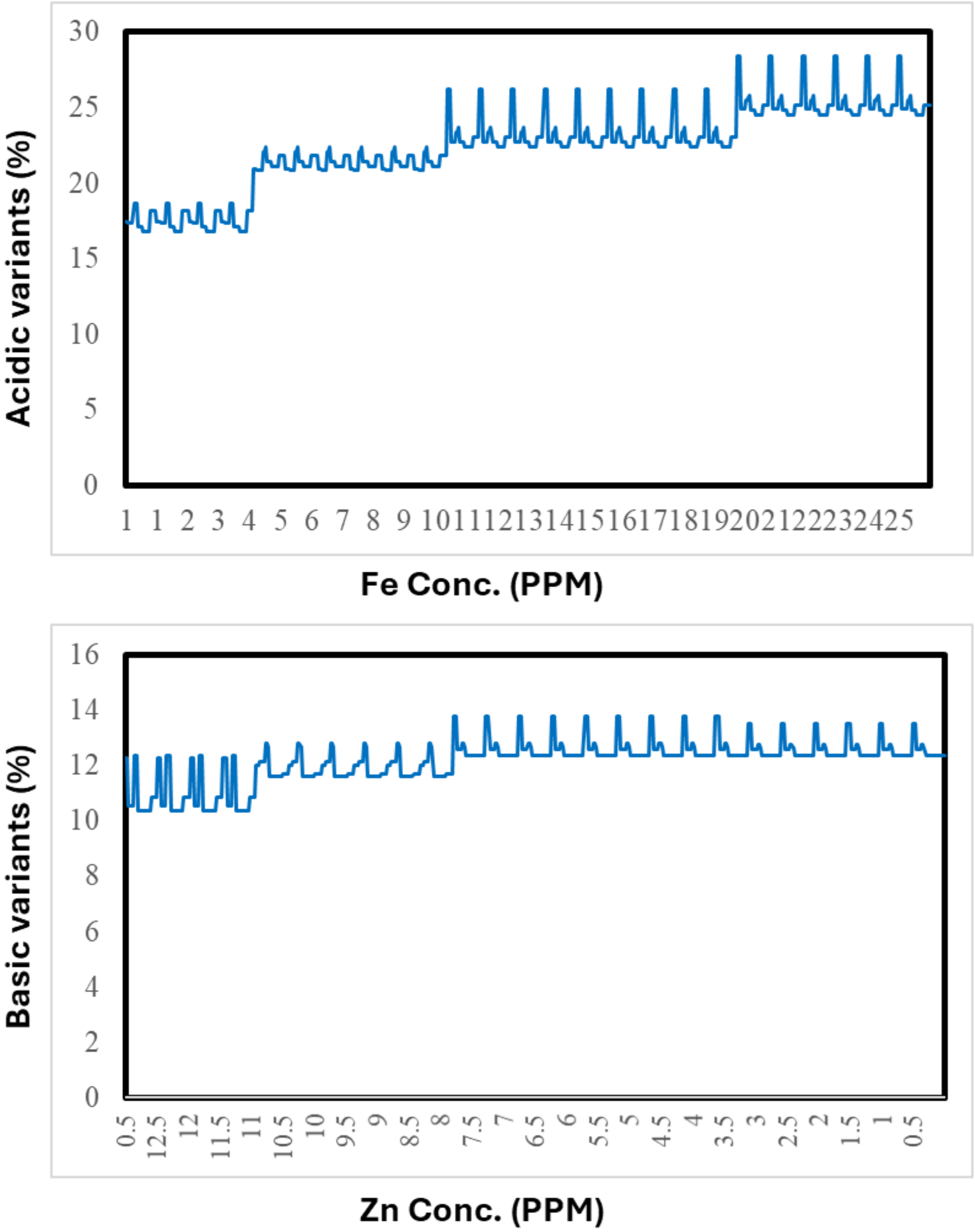

Figure S4 Plot showing relation of charge variant with Fe (Above) and Zn (Below)
